# Annotations

**Published:** 1902-10-11

**Authors:** 


### Annotations.

### Sanitary Measures and Popular Susceptibilities.

In the *Westminster Review* of this month there is a short article by Josiah Oldfield on "Fighting the Plague," in which the author endeavours to show the inutility of all sanitary measures which offend the susceptibilities of those for whose benefit they are framed. He describes how in a certain unnamed Native State in India plague appeared, and how, in deference to the recommendations of the Government, attempts were made to enforce the system then in vogue, of which segregation was the essential principle; and how, since the system was essentially opposed to the habits of the people, the whole plan broke down, the officials being outwitted and baffled at every turn by the invincible repugnance of the poor people to be removed from their own homes. Then another plan was tried. The headmen were called together, and the matter was explained to them; the city was divided into wards, and inspectors were placed over each; it was arranged that on a certain day a great holiday should be held, and that on that day, at the same time, in every quarter of the city, a great house-cleaning should take place. Disinfectants were provided free of charge, and every householder was held responsible to turn out his house, wash it, thoroughly disinfect it, and limewash it throughout. A great festival of cleansing. Then for the next eight days every room in every house had to be simultaneously fumigated for two hours with burning sulphur, and finally it was decreed, not that every case reported should be removed to camp, but that every case discovered which had not been reported should be so removed, the rest being treated at home. The result was a great success. At any rate the plague never appeared; it fled before this new festival of cleansing! This, however, does not seem to be the author's point. What he wishes to insist on is that one must go with, rather than against, the susceptibilities of the people. We will readily admit that the great, inert, immovable stupidity of the people is at the bottom of many evils, but the moral of this little tale, as we read it, is not that we should bow down and worship the ridiculous susceptibilities of the people, but, by education, remove them. The ruler of this unnamed State happened to be blessed with a ready wit which enabled him to tickle his people the right way. But it is not always so.

### The "Drayton Grange" and the "Oswestry Grange."

Nothing could be more striking than the contrast between the voyages of the two troopships, the *Drayton Grange*, from Durban to Melbourne, and the sister ship the *Oswestry Grange*, from South Africa to Southampton. On the arrival of the latter ship in port, a joint naval and military board found that, notwithstanding that she had carried 64 more men than the former, she was clean, the health of the troops was good, and there had been no overcrowding; while on the arrival of the *Drayton Grange* in Australia the condition of affairs was such as to have at once aroused the keenest and most hostile criticism, and to have led to the appointment of a Royal Commission to investigate the circumstances which led to the disasters which overtook those who sailed in her. But while the successful voyage of the *Oswestry Grange* seems to put out of court the assertions which have been made as to overcrowding of the sister ship, so far as that term relates to healthy men, we must remember that it was not long before the *Drayton Grange* became a floating hospital, and that for hospital purposes her accommodation was absolutely insufficient. It was the outbreak of measles and influenza that did the mischief. In a town population such a large proportion of the people have measles in their infancy that the disease never has the opportunity of rising to epidemic proportions among adults. But it is otherwise where the people are sparsely scattered over the land, and where the spread of infection from farm to farm is hindered by distance and by the slight amount of intercommunication which exists. Events in South Africa and among the prisoners of war from South Africa have shown how serious a disease measles may become among such a people, and probably the same influences have told to some extent among the Australians. But however the thing came about, whether from the susceptibility of the troopers, the overcrowding of the ship, or the lack of that perfection of sanitary cleanliness which alone can make such a ship healthy, no one can doubt the gravity of the state of affairs which developed when the disease once got hold. The evidence of the medical officers to the effect that more than a third of the total number of troopers on board were ill at one and the same time, shows what straits they must have been put to, and perhaps in turn explains some of the lack of discipline that has been complained of. Both measles and influenza are maladies the intensity of which is immensely aggravated by even a moderate degree of overcrowding, and both are very apt to kill by way of pneumonia.

### The Sowing of Infection.

In a most interesting Report on the Epidemic of Small-pox 1901-2, Dr. Joseph Priestley, Medical Officer of Health for Lambeth, refers, among other matters, to the extent to which small-pox was spread in London by means of workmen, who had not been protected by vaccination, being allowed by the managers of the Metropolitan Asylums Board to work on their hospitals extension at Dartford, near to the existing small-pox hospitals, which were at the time occupied by small-pox patients. From this source many cases of small-pox have occurred, Dr. Priestley says, throughout London and elsewhere. Complaints of the same nature have been made by the Kent County Council, and more than one communication on the subject has been addressed to the managers by the Local Government Board. But the doings of official bodies are absolutely inscrutable. If the rules laid down by the Local Government Board as to the sites of small-pox hospitals have any basis in fact, what are we to think of the action of the Asylums Board who, after conveying thousands of cases of small-pox 17 miles down the river in all kinds of weather, in the name of isolation, straightway surround these patients with a small army of unprotected workpeople engaged in building hospital extensions. This surely is an illogical proceeding worthy of the highest flights of officialdom!

